# Supplementary material for: Are we ready for self-sampling for cervical cancer screening? Insights from service providers and policy makers in Nepal
Source: PLOS Glob Public Health. 2025 Jan 10;5(1):e0004114. doi: 10.1371/journal.pgph.0004114 (PMC11723589; doi:10.1371/journal.pgph.0004114)
Supplement: S1 Text — (DOCX) [file pgph.0004114.s003.docx]

**Interview Guideline**

**Research Title:** **Are we ready for self-sampling for cervical cancer screening? Insights from service providers and policy makers in Nepal.**

**In-depth Interview Guideline for service providers**

The interviewer provides an overview of the research and provides the participant the PIS and the consent form. After obtaining informed consent from the participant and asking some warm up questions, the interviewer can proceeds to ask the questions using the following guidelines:

The interviewer provides a general overview of the cervical cancer screening protocol including self-sampling in the context of Nepal and proceeds to ask:

- What are your views/ perception on current state of cervical cancer screening method in the country?
- Are you aware of the cervical cancer self-sampling methods? (Since it is likely that the stakeholders may not have idea about the self-sampling kit, the researcher will probe the question to following in case of “if yes” “if no”)
  - If yes:
    - Where did you first hear about it?
    - What did you first think about it?
    - Has your perception about it changed over time?
    - Could you share your experience of working in the field of cervical cancer screening in Nepal, including self-sampling method of cervical cancer screening?
  - If- No: explain what is the self- sampling approach to cervical cancer screening?
    - Could you share your experience of working in the field of cervical cancer screening in Nepal? (Guideline, policies, paper work for approval)
- How do you perceive self- sampling method of cervical cancer screening to the women of Nepal? (Perception- feasible, acceptable to whom, where; thoughts on cervical cancer self-sampling methods, how was the experience)

***Follow up questions:***

- - Do you think such new method or plan could benefit the population?
  - Opportunities and/or challenges regarding cervical cancer screening including self-sampling approach to cervical cancer screening
- What could be the benefit or harm of self-sampling approach to cervical cancer screening?
- Will government or private hospitals and health care providers prioritize self-sampling of cervical cancer screening? why or why not?
- Could you share with us, in your opinion what could be potential challenges for women to utilising the self-sampling of cervical cancer screening service provided through government or private hospitals?
- How can we overcome the barriers and promote facilitators for adopting the method? What could be the role of service providers to facilitate the adoption of self-sampling approach and overcome the barriers?
- How would you suggest introducing the self-sampling kit to the policy/population?

[For online service providers]

- Could you share your experience and knowledge self-sampling/ self- testing/ medicines/ home collection services/ delivery of SRH services?
- How has the response been so far for delivering such services in Kathmandu valley?
- Could you explain more regarding the challenges and barriers to home collection services?
- How do you perceive self- sampling method of cervical cancer screening to the women of Nepal? (Perception on adopting self- sampling method in Nepal)
- What would be the barriers and facilitators for adopting the self - sampling method?
- How would you suggest introducing the self-sampling kit to the population?
- Will you be able to provide self-sampling service for cervical cancer screening if provisioned?

**Key-Informant Interview Guideline for policy makers**

The interviewer provides an overview of the research and provides the participant the PIS and the consent form. After obtaining informed consent from the participant and asking some warm up questions, the interviewer can proceeds to ask the questions using the following guidelines:

The interviewer provides a general overview of the self-sampling of cervical cancer screening in the context of Nepal and proceeds to ask:

- What are your views/ perception on current state of cervical cancer screening method in the country?
- Are you aware of the cervical cancer self-sampling methods? (Since it is likely that the stakeholders may not have idea about the self-sampling kit, the researcher will probe the question to following in case of “if yes” “if no”)
  - If yes:
    - Where did you first hear about it?
    - What did you first think about it?
    - Has your perception about it changed over time?
    - Could you share your experience of working in the field of cervical cancer screening in Nepal, including self-sampling method of cervical cancer screening?
  - If- No: explain what is the self- sampling approach to cervical cancer screening?
    - Could you share your experience of working in the field of cervical cancer screening in Nepal? (Guideline, policies, paper work for approval)
- How do you perceive self- sampling method of cervical cancer screening to the women of Nepal? (Perception- feasible, acceptable to whom, where; thoughts on cervical cancer self-sampling methods, how was the experience)

***Follow up questions:***

- - Do you think such new method or plan could benefit the population?
  - Opportunities and/or challenges regarding cervical cancer screening including self-sampling approach to cervical cancer screening
- Could you share with us, in your opinion what could be the potential opportunities for women to utilising the self-sampling of cervical cancer provided through government? (Existing policy mechanism, learnings from other countries)
- Could you share with us, in your opinion could be the potential challenges for women to utilising the self-sampling of cervical cancer provided by government? (socio-cultural context, financing of self-sampling approach, feasibility, acceptability)
- What are the existing policy barriers to facilitate self-sampling of cervical cancer screening?
- How do we overcome the barriers and promote facilitators for adopting the method?
- How would you suggest introducing the self-sampling kit to the policy/population?
