# Supplementary material for: Are we ready for self-sampling for cervical cancer screening? Insights from service providers and policy makers in Nepal
Source: PLOS Glob Public Health. 2025 Jan 10;5(1):e0004114. doi: 10.1371/journal.pgph.0004114 (PMC11723589; doi:10.1371/journal.pgph.0004114)
Supplement: Attachment — Submitted filename: Re_Reviewers_response May 20, 2024.doc [file pgph.0004114.s004.doc]

I'd like to acknowledge the authors’ efforts in raising awareness about the importance of self-sampling to improve the acceptance of self-sampling for cervical cancer screening in Nepal. Unfortunately, some areas have not been thoroughly addressed, and there are still some confusing segments demanding review for improvement.

**MAJOR CONCERNS:**

1. **Line 101-102**: The authors mentioned settings including Kathmandu, Bhaktapur, and Lalitpur districts of Nepal. Unfortunately, only one was elaborated on for the reason of their choice.
2. Worryingly, the authors further mentioned, “Kathmandu Valley was purposively selected for the study sites due to the presence of tertiary level health facilities, digital/online service providers, and federal-level policymakers who are based in Kathmandu Valley.” This poses a significant concern as it introduces bias. This raises questions about the minds and opinions of girls in rural and low-resource settings. Unfortunately, the study is titled “…in Nepal,” which does not represent important opinions as required.

In other words, the statement suggests that the data cannot mirror the opinions of non-educated and/or rural women. What about awareness in this group?

3. Furthermore, in Lines 119-138, the statement, “Participants were provided with an information sheet detailing the study's goals and ethical considerations. Study participants were also given information about the self-sampling approach and how to use the self-sampling kit, and the kit was demonstrated during the interview,” raises concern due to the information released to the participants. How the authors would be able to filter the authenticity of their statements from personal thoughts is a big question.
4. **Line 220-223**; One of the points raised, “So the knowledge about screening is the main reason why its status is low. Most of the patients go for treatments only with a doctor’s advice or after their visit to a gynecologist. Based on my knowledge, the patients who visit solely for screening are the least,” raises more concern regarding personal opinions versus reality. A reasonable and inquisitive mind would ask how the opinions of uneducated countrywomen, as captured in this statement, actually represent the true scenario.
5. In addition to the above, the authors further caused confusion by mixing “self-sampling” with “self-screening” as reflected in their text;
a. **Line 191-193 and some other areas,** “The people in urban cities are mostly aware and educated. Not only in Kathmandu valley but also in other urban cities like- Narayanghat, Butwal, and Pokhara. If we promote it and inform about this new method then I believe people would perform self-screening easily”.
b. And **Line 278-280:** “The main thing for promotion is to spread public awareness of this cervical cancer test kit among people about how to do screening at home, who can test it, where to send the sample”. (Online service provider-1)”
6. **Line 215-216**: “a lack of privacy during pelvic examinations are highlighted as barriers by health care providers…” is not clear. One would like to know if the authors meant that sampling dor the screening is done in Nepal without proper enclosure.
7. **Line 263-265**; The statement “There may be cases where they find it difficult to take their own samples, they may be skeptical whether the sample they take will be good enough or not, or they might fear that what they are doing is probably not right”. (Representative DOHS-3) suggests that this kind of statement demands hearing some of these women directly......it raises concerns of accuracy, or honesty, personal emotion could affect the responses!!!
8. There is a lot of info glaringly missing from the rural part as evidenced even in the care providers' response, “Online purchase of self-sampling kit is possible in urban areas. If the client pays the transportation charge, we will provide it. **We don't know the feasibility in the villages.** But in the city, women do not shy away to buy the medical product online from our experience”.
9. **Line 377;** The authors mentioned, “This is the first study to explore the feasibility and its acceptability of HPV self- sampling in Nepal with service providers.” Unfortunately, Ref 24 suggesting the same group of authors had earlier conducted this kind of study before and proved the authors wrong. This is worrying!
10. **ABSTRACT:**

The statement reading “an unclear screening process” is unclear and therefore requires correction to maintain good flow.

11. All abbreviations using apostrophes such as “It's” should be written out in full in formal writing.

**12**. Several sentences taken from the literature are not properly cited, which is a grave scientific violation. A few of these are listed below:

a. Line 63: “The World Health Organization (WHO) recommends a well-organized system for effective screening, diagnosis, treatment, and follow-up of cervical cancer.”

b. Line 69: “The initial target was to screen at least 50% of women in the age group of 30–60 years, by 2015, revised in 2017 to 70%.” If this is a statement from the same authors as the subsequent sentence, both sentences should have been merged.

c. And many others.

1. Line 73: The use of the connector “However” within 2-3 sentences would benefit from being changed to words like “Unfortunately” to maintain good flow. “However, a systematic review and meta-analysis suggests low cervical cancer screening where only 17% of the cervical cancer screening occur in hospitals while just 16% of the screening takes place in the community using the Pap Smear test for cervical cancer screening in Nepal (7).”
2. Many grammatical errors and punctuation issues were noted.

**GENERAL COMMENTS**

I think the authors failed to clarify the difference between self-sampling as just a part of the screening process and self-sampling as the entire screening method. Their erratic reporting made the concept difficult to understand.

Of most significance is the introduction of bias by informing the participants about the project in detail. One does not need to be a trained psychologist to see that the opinions recorded in the study may not accurately reflect real scenarios. In fact, one of the statements mentioned a lack of knowledge among participants.

The authors did not provide a good rationale for their choice of study settings and how these settings represent the entire country. Although the title mentions Nepal, only 20 opinions were recorded, which is insufficient to represent the whole country. These opinions are partially disconnected from reality and fail to include rural scenarios.

Thank you.
