# Supplementary material for: Are we ready for self-sampling for cervical cancer screening? Insights from service providers and policy makers in Nepal
Source: PLOS Glob Public Health. 2025 Jan 10;5(1):e0004114. doi: 10.1371/journal.pgph.0004114 (PMC11723589; doi:10.1371/journal.pgph.0004114)
Supplement: Attachment — Submitted filename: Response to Reviewers.docx [file pgph.0004114.s005.docx]

Title: Are we ready for self-sampling for cervical cancer screening? Insights from service providers and policy makers in Nepal

**Response to Reviewer**

Dear editor and reviewer, thank you very much for taking time to review our manuscript. The feedback from the reviewer has been helpful in refining the manuscript. As advised in the revision letter, we have revised the manuscript and provided point to point details below about the revision. We have addressed all the comments provided by the academic editor and reviewer in our revised manuscript. We have also uploaded a track change version and clean version as suggested. Please find point-to-point details below. Should you have any questions or concerns, please feel free to reach out to me.

On behalf of co-authors,

Saki Thapa

saki.thapa@gmail.com

| **Editor’s/Reviewer’s comments** | **Response** | **Corrected page#, and lines#** |
| --- | --- | --- |
| **Reviewer #1** | | |
| 1. Line 101-102: The authors mentioned settings including Kathmandu, Bhaktapur, and Lalitpur districts of Nepal. Unfortunately, only one was elaborated on for the reason of their choice. | Thank you very much for your remarks.  Kathmandu Valley includes Kathmandu district, Bhaktapur district and Lalitpur district. The manuscript says Kathmandu valley as setting and not just Kathmandu district. | Page #7, Lines # 131-132 |
| 2. Worryingly, the authors further mentioned, “Kathmandu Valley was purposively selected for the study sites due to the presence of tertiary level health facilities, digital/online service providers, and federal-level policymakers who are based in Kathmandu Valley.” This poses a significant concern as it introduces bias. This raises questions about the minds and opinions of girls in rural and low-resource settings. Unfortunately, the study is titled “…in Nepal,” which does not represent important opinions as required.   In other words, the statement suggests that the data cannot mirror the opinions of non-educated and/or rural women. What about awareness in this group? | This study adopts purposive sampling and the purpose for adopting purposive sampling in site selection was to understand and explore the readiness of service provider for self-sampling of cervical cancer screening in the area where the implementation of self-sampling is evident for information rich cases.  This has also been clearly mentioned by Palinkas et. al which is cited by 14,862 articles. Since, the study is qualitative, random sampling is not a right sampling design for this study. | Page #7, Lines #136-142 |
| 3. Furthermore, in Lines 119-138, the statement, “Participants were provided with an information sheet detailing the study's goals and ethical considerations. Study participants were also given information about the self-sampling approach and how to use the self-sampling kit, and the kit was demonstrated during the interview,” raises concern due to the information released to the participants. How the authors would be able to filter the authenticity of their statements from personal thoughts is a big question. | Yes we agree. The statement Study participants were also given information about the self-sampling approach and how to use the self-sampling kit, and the kit was demonstrated during the interview" was irrelevant statement for this study. This sentence has been deleted from the section. The participants were provided with information sheet detailing the study's goal and ethical consideration for participant's clarity on the research before making the decision to participate. Thank you for highlighting this major error. | Page #8, Lines #161-162 |
| 4. Line 220-223; One of the points raised, “So the knowledge about screening is the main reason why its status is low. Most of the patients go for treatments only with a doctor’s advice or after their visit to a gynecologist. Based on my knowledge, the patients who visit solely for screening are the least,” raises more concern regarding personal opinions versus reality. A reasonable and inquisitive mind would ask how the opinions of uneducated countrywomen, as captured in this statement, actually represent the true scenario.s | The response is from the policymaker who speaks about self-sampling in urban area among urban women and girls and not about "uneducated country women". The qualitative research needs to trust the respondent's experience, which this research does. |  |
| 5. In addition to the above, the authors further caused confusion by mixing “self-sampling” with “self-screening” as reflected in their text; a. Line 191-193 and some other areas, “The people in urban cities are mostly aware and educated. Not only in Kathmandu valley but also in other urban cities like- Narayanghat, Butwal, and Pokhara. If we promote it and inform about this new method then I believe people would perform self-screening easily”. b. And Line 278-280: “The main thing for promotion is to spread public awareness of this cervical cancer test kit among people about how to do screening at home, who can test it, where to send the sample”. (Online service provider-1)” | Thank you for the heads up regarding the ambiguity. We have used "self-sampling" across the manuscript for uniformity and clarity. |  |
| 6. Line 215-216: “a lack of privacy during pelvic examinations are highlighted as barriers by health care providers…” is not clear. One would like to know if the authors meant that sampling dor the screening is done in Nepal without proper enclosure. | The phrase "a lack of privacy during pelvic examination" has been removed. | Page# 18, Line # 311 |
| 7. Line 263-265; The statement “There may be cases where they find it difficult to take their own samples, they may be skeptical whether the sample they take will be good enough or not, or they might fear that what they are doing is probably not right”. (Representative DOHS-3) suggests that this kind of statement demands hearing some of these women directly......it raises concerns of accuracy, or honesty, personal emotion could affect the responses!!! | This is the limitation of the study and this has been mentioned in the limitation section of the study. However, in qualitative study, these anecdotes are equally important. The study focuses on information, knowledge and beliefs of service providers and policy makers – we did not interview women for this study. |  |
| 8. There is a lot of info glaringly missing from the rural part as evidenced even in the care providers' response, “Online purchase of self-sampling kit is possible in urban areas. If the client pays the transportation charge, we will provide it. We don't know the feasibility in the villages. But in the city, women do not shy away to buy the medical product online from our experience”. | This study focuses on the urban settings and not on rural settings. Thus, the data regarding the feasibility in the rural areas are not explored as part of this study, which is a limitation and this has been mentioned in the limitations as well as recommended for future studies. Online service provision is very new in Nepal and currently service delivery in rural areas would still in the main require a program of outreach camps. |  |
| 9. Line 377; The authors mentioned, “This is the first study to explore the feasibility and its acceptability of HPV self- sampling in Nepal with service providers.” Unfortunately, Ref 24 suggesting the same group of authors had earlier conducted this kind of study before and proved the authors wrong. This is worrying! | Thank you for flagging this. The previous article explores the feasibility of self-sampling among women and girls while this study studies the feasibility AND acceptability of services among service providers and policy makers. This study is follow up study to bridge the limitation of previous study conducted. Your feedback is important and thus we do not claim to be first article and the sentence has been revised as "This study explores the feasibility and acceptability of HPV self-sampling in Nepal among service providers and policy makers." | Page # 27, Line #506 |
| 10. ABSTRACT:   The statement reading “an unclear screening process” is unclear and therefore requires correction to maintain good flow. | This has been edited to "unclear information regarding self-sampling process among women and girls". | Page #3, Line #47-49 |
| 11. All abbreviations using apostrophes such as “It's” should be written out in full in formal writing. | The comment has been addressed and changes have been made as suggested. Thank you. | Page #3, Line #55  Page #17, Line #303  Page #24, Line #446  Page #25, Line #476  Page # 25, Line #480 |
| 12. Several sentences taken from the literature are not properly cited, which is a grave scientific violation. A few of these are listed below:   a. Line 63: “The World Health Organization (WHO) recommends a well-organized system for effective screening, diagnosis, treatment, and follow-up of cervical cancer.”  b. Line 69: “The initial target was to screen at least 50% of women in the age group of 30–60 years, by 2015, revised in 2017 to 70%.” If this is a statement from the same authors as the subsequent sentence, both sentences should have been merged. | Appropriate citations have been provided as suggested. | Page #4, Lines 72-76  Page #5, Line 84-85 |
| a. And many others. b. Line 73: The use of the connector “However” within 2-3 sentences would benefit from being changed to words like “Unfortunately” to maintain good flow. “However, a systematic review and meta-analysis suggests low cervical cancer screening where only 17% of the cervical cancer screening occur in hospitals while just 16% of the screening takes place in the community using the Pap Smear test for cervical cancer screening in Nepal (7).” c. Many grammatical errors and punctuation issues were noted. | Thank you for flagging it up.  The language, grammatical errors and punctuation has been reviewed throughout the manuscript and well addressed. |  |
| **Reviewer #2** | | |
| 1. Lines 55-57: Would you consider referencing GLOBOCAN 2022 and check if cancer of the cervix is not currently in the third place globally in women? | Thank you for the heads up. We have checked the GLOBOCAN 2022 and edited the cervical cancer rank from top fourth to top ten with the references. | Page #4, line #62-64 |
| 2. Line 57: Is Nepal a low- or middle-income country? | According to New World Bank country classifications by income level: 2022-2023, updated date: JULY 01, 2022, Nepal is lower-middle income country.  Link: <https://blogs.worldbank.org/en/opendata/new-world-bank-group-country-classifications-income-level-fy24> |  |
| 3. Lines 59 – 60: You need to briefly discuss the link between the cancer of the cervix and human papillomavirus infection. Also, what is the comparator globally i.e., what is the percentage of women harbouring HPV globally. | The link between the cancer of the cervix and HPV has been added as “If left untreated, a persistent HPV infection of the cervix causes 95% of cervical cancers: in the manuscript with the reference. | Page #4, Line #69-70 |
| 4. Lines 63 - 65: A brief mention of WHO’s position on HPV testing might be helpful. Further, its strategy is to screen 70% by 2030. Since you are writing for a global audience, it helps to bring in global context | This has been added in the manuscript as “The World Health Organization (WHO) recommends women should be screened for cervical cancer every 5-10 years, starting at age 30 while screening should be undertaken every 3 years for women living with HIV, starting at age 25.” | Page #4, Lines #72 - 76 |
| 5. Line 67: What sort of age groups should be screened according to CCSP i.e., lower and upper age boundaries? | The initial target of CCSP was to screen at least 50% of women in the age group of 30–60 years. This has been mentioned in the manuscript. | Page #5, Lines #84-85. |
| 6. Line 71: “…Nepal lacks a national screening program for cervical cancer…..” but in line 67 you mention CCSP. Is this not contradictory? | A systematic review and meta-analysis study by Shrestha on Cervical cancer screening utilization, and associated factors, in Nepal published on September 2022, mentioned that there is no national screening programme in Nepal until now. A study done by Dangal (a study on implementation of cervical cancer prevention and screening across five tertiary hospitals in Nepal and its policy implications published on January 2024) mentioned that cervical cancer screening in Nepal is available at both the population level and within hospitals. While population-based outreach health camps are done in communities, primarily organized by hospitals in collaboration with government and non-government organizations and tertiary care centers provide cervical cancer screening services in Nepal as outpatient services. There is no structured population based national cancer screening program in Nepal. | Page #5, Lines #87- 94 |
| 7. What is the reference, please? | The reference and details has been added as following in the manuscript- “A systematic review and meta-analysis study by Shrestha on Cervical cancer screening utilization, and associated factors, in Nepal published on September 2022, and A study done by Dangal (a study on implementation of cervical cancer prevention and screening across five tertiary hospitals in Nepal and its policy implications published on January 2024) mentioned that cervical cancer screening in Nepal | Page #5, Lines #87-84 |
| 8. Line 407: There is mention of out-of-pocket expenditure. What is the country’s funding program for medical expenses supposed to be like? | This has been explained in the discussion section by adding specialized services are covered financially under health insurance program | Page #28, Lines #541- 543 |
| 9. Is there any study you could reference that discusses reasons for not taking up the current screening methodologies by women in Nepal? | This has been explained in the discussion section by adding “Similar to these findings, other studies conducted in different countries to explore feasibility and acceptability with end users showed that women are willing to accept the self-sampling approach to cervical cancer screening compared with clinician-based cervical cancer screening in the clinical settings due to social and cultural barriers, inconvenience and confidential barriers”. | Page #27, Lines #524-528 |
| 10. Did you pilot the instruments? It might be helpful to add that information here if you did? | Yes, we piloted the interview guide among four participants but we did not include it in analysis of the study. | Page # 8, Lines  #167-168 |
| 11. How many female participants were in your study? Attitudes to self-sampling might differ by gender. | The participants of this study are health care providers and policy makers. Thus female participation in this study is not relevant for self-sampling experience of participants. We have added a Table 2, Characteristic of the participant of the study which includes gender ( Male= 7, and Female=13) occupations and affiliation. | Page #13, Line #226 |
| 12. It would have been interesting to include the views of the laboratory technologists because they are on the receiving end, and they are the ones who analyse the samples. Hence, they are in a better position to comment about correctness and quality of self-collected samples. | Thank you for this important suggestion. The research team will be more careful in future considering incorporating such important respondent in our research. |  |
| 13. Comment on whether women are charged for cervical cancer screening | Nepal has adopted the 'screen-and-treat' approach to cervical cancer prevention, utilizing the Visual Inspection with Acetic Acid (VIA) test. VIA tests are available free of charge at government health institutions, including hospitals and health posts, in 64 of Nepal's 77 districts for women aged 30-60 years. People pay fees for Pap smear tests in the locations where the necessary technical and laboratory facilities exist. | Page #5, Lines #90-94 |
| 14. Is self-sampling already available? | Self-Sampling is relatively new concept for Nepal. To our knowledge there are no services available for self-sampling in general population available in Nepal. There are ongoing feasibility studies to support piloting and scale up of self-sampling for cervical cancer screening. | Page# 6, Lines #111-113 |
| 15. Is this process not a deterrent to cervical cancer screening? • Who is getting it? | According to WHO recommends screening with HPV DNA testing should start at the age of 30 years with regular testing every 5–10 years for women. In contrast, women with HIV should begin screening at 25 years, with regular screening every 3–5 years. | Page#6, Lines #114- 116 |
| 16. What are the limitations and strengths of this project? | The limitation and strength of this study has been mentioned in the discussion section and separately in limitation sections as well. The limitation section includes- The study provides valuable insights into the feasibility and acceptability of HPV self-sampling for cervical cancer screening among health care providers and service providers at federal level but does not explore the acceptability and feasibility of self-sampling approach among local government and service providers, which is one of the limitations of this study. Thus, this study recommends further research to capture perspective of local service providers and policy makers, pilot study to measure the convenience of self-sampling approach among women and girls, and assess the cost-effectiveness and accuracy of self-sampling in Nepal which is crucial to guide national programs and policies for prioritization and effective implementation of self-sampling approach in cervical cancer screening .” | Page# 30, Lines #579- 587 |
| **Reviewer #3** | | |
| 1. Presenting the perspectives on, or the definitions of “acceptability” and “feasibility” used in the study would allow readers to grasp how the results are interpreted, as these terms are widely used in global health and implementation research. | Self- sampling is seen as acceptable in terms of privacy and confidentiality and easy to use for women. The self-sampling is seen feasible mostly in terms of delivery of services, and level of education mostly in Kathmandu valley. Details has been addressed in the manuscript in the result sections with supporting excerpts. | Page#14, Lines # 229- 231  Page 15, Lines #-260-264 |
| 2. In the first paragraph, although HPV-16/18 infection is mentioned, there is no explanation or reference to its role as the main cause of cervical cancer. Introducing this idea briefly would facilitate readability. Additionally, acronyms such as HPV should be spelled out in full the first time they are used. | The comment has been duly addressed with adding “If left untreated, a persistent HPV infection of the cervix causes 95% of cervical cancers.” in the manuscript. | Pages #4, Lines #69-70 |
| 3. A reference to the WHO Global Strategy for Cervical Cancer Elimination would be beneficial, particularly when mentioning the target for women screened according to the national guidelines, since it is in line with the WHO global strategy. | A reference to the WHO Global Strategy for Cervical Cancer Elimination has been addressed in the manuscript | Page# 4, Lines #62- 64 |
| 4. In line 84, ("the Government of Nepal highlights the challenge of a technology-based programme but emphasizes the importance of self-sampling in reaching larger populations in the reference manual for Cervical Cancer Screening in Nepal 2015”), more context could be included) so that the reader understands what specific challenges the Government of Nepal is referring to. | This has been addressed and explained as “In the context of Nepal, the Government of Nepal emphasizes the importance of self-sampling in reaching larger populations in the reference manual for Cervical Cancer Screening in Nepal 2015 but highlights potential difficulty of follow up with treatment for test-positive clients. Despite the existence of feasibility studies for HPV self-sampling methods, the promotion of self-sampling has not been extensively incorporated into the policies or programs of Nepal.” | Page#6, Lines #110-113 |
| 5. The reference to purposive sampling sounds repetitive (it is mentioned twice - lines 98 and 106, though it is not needed in the study design paragraph). | The line deleted- “Purposive sampling was implemented and participants with knowledge and experiences working on cervical cancer were included in the study” from the manuscript. | Pages # 6- 7, Lines #127-129 |
| 6. More details on the criteria used to select the study settings would be beneficial: why are tertiary level health facilities important within the context of this study? | In Nepal, mostly tertiary hospitals provide cervical cancer screening services. | Page# 7. Lines #133- 136 |
| 7. What are digital/online delivery services (lines 109, 128-129)? | The online delivery services that we interviewed are registered under Nepal company act, and receive order from general population via online i.e from social media , mobile application and their websites. |  |
| 8. Lines 115-116: “Purposive sampling was adopted for data saturation”. Can the authors explain what they mean here? | The data saturation was deleted from the manuscript. The purposive sampling is extensively used sampling method in qualitative study which involves identification and selection of information rich and experienced participants to provide information to help answer the research question and objective. Proper citation has been made to support our case. | Page #8, Line #158 |
| 9. Ethical considerations usually go in a separate specific subsection within the methods section. Beyond the formatting aspect, I recommend not mixing this information with other content (see lines 112-122). | There is no specific format regarding ethical considerations section for PLOS global health. However, We have separated the ethical consideration section in the manuscript. | Page #8, Lines #153 |
| 10. (Lines 201-203): “However, some of the participants of this study suggest that self-sampling is not feasible and acceptable in the context of Nepal, preferring to support screening by health care providers, and anticipating potentially low participation from the service users”. Would it be possible to present the reasons expressed by these participants not to perceive it acceptable/feasible? | The reason has been explained in the discussion section mainly due to cost and accuracy which has been explained in the manuscript. | Page # 28, Lines #543-545  Lines #552-553 |
| 11. Line 224: no need to spell out IDI again. | The comment has been addressed and short form IDI has been used. |  |
| 12. There is a subsection named “Inclusive policy and national strategies” that does not appear in the list of themes identified through the thematic analysis, which seems confusing at this point. While qualitative analytical approaches can identify emerging themes outside the study's initial objectives, it would be beneficial to contextualize the reader when introducing unexpected findings. | The Inclusive policy and national strategies is sub-theme under theme “Feasibility facilitators for self-sampling” of Table 1. There was formatting error and error in the infographics which has been corrected. | Page#13, Line# 218 |
| 13. Discussion • Lines 381-383: “Convenience, confidentiality, and a low level of embarrassment have been identified as key characteristics of adopting self-sampling among the participants.” This sentence seems not to be aligned with the results presented in the previous section. Can the authors provide some clarifications? | The information has been added in the result section by adding “Similar to these findings, other studies conducted in different countries to explore feasibility and acceptability with end users showed that women are willing to accept the self-sampling approach to cervical cancer screening compared with clinician-based cervical cancer screening in the clinical settings due to social and cultural barriers, inconvenience and confidential barriers.” | Page#27, Lines #524-529 |
| 14. Lines 387-390: “…a few participants of this study were sceptical about the acceptability of self-sampling method by young girls and women, the myths and misconceptions regarding self-sampling approach among certain sections of society, particularly those of low literacy and poor socio-economic status”. There’s no mention of myths and misconceptions in the results, thus, it seems this is an assertion not supported by reported data. | The feedback has been addressed and 'myths and misconception' has been deleted. | Page#27, Line #519- 520 |
|  | | |
