# Supplementary material for: Are we ready for self-sampling for cervical cancer screening? Insights from service providers and policy makers in Nepal
Source: PLOS Glob Public Health. 2025 Jan 10;5(1):e0004114. doi: 10.1371/journal.pgph.0004114 (PMC11723589; doi:10.1371/journal.pgph.0004114)
Supplement: Attachment — Submitted filename: Response to Reviewers.docx [file pgph.0004114.s006.docx]

Title: Are we ready for self-sampling for cervical cancer screening? Insights from service providers and policy makers in Nepal

**Response to Reviewer**

**Dear Reviewer,**

Thank you very much for taking the time to thoroughly review our manuscript. Your insightful and detailed feedback has been invaluable in helping us refine and strengthen our work.

As per your suggestions and the revision letter, we have carefully revised the manuscript, addressing all comments from you. Below, we have provided a point-by-point response to each comment. Additionally, we have uploaded both a track change version and a clean version of the revised manuscript for your review.

We sincerely appreciate your important review and guidance throughout this process. If you have any further questions or concerns, please do not hesitate to reach out.

On behalf of co-authors,

Saki Thapa

saki.thapa@gmail.com

| **Editor’s/Reviewer’s comments** | **Response** | **Corrected page#, and lines#** |
| --- | --- | --- |
| **Reviewer #2** | | |
| 1. You need to be consistent about categories of participants. The way that I understand it is that you have: (i) healthcare providers (nurses and gynaecologists), (ii) service providers (online services) and (iii) policymakers. This is not always clear as you switch between two and three. | This comment has been addressed and the terminologies health care providers, online service providers and policymakers has been consistently used across the manuscript. The online service providers are not health care providers and cannot be categorized otherwise. |  |
| 2. You have not included education/literacy as a barrier to self-sampling | There is a specific sub-theme **Low level of Knowledge and Information** (line 287-294) dedicated regarding low literacy/education level Service providers noted that women in the community have a low level of awareness about HPV and cervical cancer, which is a significant barrier to cervical cancer screening in Nepal. Additionally, they highlighted that women have very limited knowledge about the self-sampling method. This finding aligns with similar studies conducted in various settings, including Nepal (Adewumi et al., 2022; Darj et al., 2019; Kirubarajan et al., 2021). | Page 16-17, Lines # 287-294 |
| 3. Further, you have not discussed understanding sampling processes as a barrier. | Thank you for your comment. The clarity in sampling process has been discussed in the line 524-538 of the manuscript. | Pages 27-28, Line #524- 538 |
| 4. What is the strength of the study? | The study provides valuable insights into the feasibility and acceptability of HPV self-sampling for cervical cancer screening among healthcare providers and policymakers at the federal level, addressing a modern approach that could enhance accessibility for women. By offering specific recommendations for future research and aiming to inform national programs, the study contributes significantly to improving cervical cancer prevention and early detection efforts in Nepal. |  |
| 5. The recommendation is under limitations | Thank you for your comment regarding the recommendation being placed under the limitations section. To address this, we have updated the section title to "Limitations and Recommendations of the Study" to make it more coherent and reflective of the content | Page 28, Line #539 |
| 6. From your understanding, how would government subsidies this method for an average hospital attendee. | From my understanding, government subsidies for the self-sampling approach to cervical cancer screening could be implemented by providing free or low-cost self-sampling kits at public health facilities. Additionally, the government could collaborate with local healthcare providers to distribute these kits through community outreach programs. Subsidies could also cover the costs of processing the samples and delivering the results, making the entire screening process affordable and accessible to average hospital attendees, especially in underserved areas. |  |
| 7. Also, make some mention of how gynaecological services are paid for in Nepal. | In Nepal, gynaecological services are available through both public and private healthcare systems. In government hospitals, basic gynaecological services, including consultations and certain treatments, are often subsidized or provided free of charge. However, more advanced services, such as specialized tests or surgeries, may require out-of-pocket payments, particularly in private healthcare settings. Many individuals, especially in rural areas, rely on government facilities due to the high costs of private healthcare services. |  |
| 8. Please pay attention to grammar | Thank you for your feedback. We have reviewed the manuscript carefully to ensure proper grammar throughout the text. Your attention to detail is appreciated, and we aimed to enhance the clarity and professionalism of our writing. |  |
| **Specific Comments:**   9. Line 40: “Screening” should be “sampling”. | Thank you for flagging this. We have corrected screening to sampling. | Page # 2, Line #42 |
| 10. Line 43: “Women and girls” does this refer to study’s female participants i.e. study population or is it referring to users users? | The term 'women and girls' in this context refers to the intended users of the cervical cancer screening self-sampling kits. They are the target audience or beneficiaries of the service | Page #3, Line #45 |
| 11. Line 61: Papilloma virus is one word “papillomavirus” | The comment has been addressed and changes have been made as suggested. Thank you. | Page #3, Line #63 |
| 12. Line 66: Reference? | Thank you. Appropriate citations have been provided as suggested. | Page #4, Lines 66-68 |
| 13. Line 69: use “WHO” instead of the fully spelled out World Health Organization. | Thank you. We have addressed it by replacing 'World Health Organization' with 'WHO' in the manuscript." | Page #4, Line 71 |
| 14. Line 71: Do not use capital letters for the first letter for Cervical Intraepithelial Neoplasia | Thank you for your feedback. We have corrected the text to lowercase letters for 'cervical intraepithelial neoplasia' in the manuscript | Page #4, Line #73 |
| 15. Line 74: Do not use capital letters for the first letter for Cervical Cancer Screening and Prevention. | Thank you for your comment. We have made the necessary changes to ensure that cervical cancer screening and prevention' is written in lowercase throughout the manuscript | Page #4, Line #76 |
| 16. Lines: 82-83: Not understandable. | Thank you for your feedback. I have revised the sentence for clarity. The updated version now reads: 'In Nepal, cervical cancer screening is offered through two main approaches: population-based outreach health camps in communities, which are organized by hospitals in collaboration with government and non-government organizations, and outpatient clinical services, which are mainly provided by tertiary care centers. | Page #5, Lines #84 - 87 |
| 17. Lines 82-88: Very long sentence. Break it down into shorter sentences. | Thank you for your observation regarding the sentence length. We have revised the text to break it into shorter, clearer sentences for improved readability. The updated version is as follows:  VIA test that the Government of Nepal has implemented for cervical cancer prevention following WHO recommendations (6, 7) are available for free at government health institutions in 64 out of 77 districts for women aged 30–60 years. While people pay fees for Pap smear in locations where the necessary technical and laboratory facilities exist, a systematic review and meta-analysis suggests low cervical cancer screening uptake with 17% of the cervical cancer screening occurring in hospitals and 16% of the screening take place at the community (9). | Page #5, Lines #88 - 94 |
| 18. Lines 94-97: Already alluded to in lines 64-66. | The line – “According to WHO recommendations, women should begin HPV DNA testing at age 30, with regular screenings every 5–10 years. In contrast, women with HIV should start screening at age 25, with regular screenings every 3–5 years” was deleted. | Page #5, Lines #100-103 |
| 19. Line 99: Do not use capital letters for the first letter for Cervical Cancer Screening. | Thank you for your comment. We have made the necessary changes to ensure that 'cervical cancer screening' is written in lowercase throughout the manuscript | Page #6, Line #105 |
| 20. Line 127: “18 years and above” were those patients? | Thank you for your comment. The study participants included healthcare providers and policymakers aged 18 years and above, with experience and expertise in cervical cancer screening, self-sampling, treatment, and/or policymaking. | Page #7, Lines #140 |
| 21. Lines 119-124: Should go under sampling i.e., the section below | Thank you for your observation. We have moved the information regarding the study sampling under sampling section for better clarity and alignment with the study structure. | Page #7, Lines #134- 139 |
| 22. Lines 139-141: Repetition. Further, they do not belong in this section. | Thank you for pointing out the repetition and the placement issue. We have revised the manuscript to remove the repetitive content to improve clarity and structure. | Page #8, Lines #152-154 |
| 23. Line 226: “Alternative to being sampled as a healthcare provider.” | This has been revised as " Most of the study participants of this study identify self-sampling as an acceptable and potential alternative approach for cervical cancer screening in Nepal." | Page # 14, Line #238-240 |
| 24. Line 257: “Support sampling by” | Deleted “ to support “ to make the sentences clear. | Page #16, Line 271 |
| 25. Lines 281-282: Repetition. Delete. | Thank you for your comment. We have removed the repetitive content – “Health care providers and policy makers report that the low level of cervical cancer screening is because of lack of knowledge and awareness among women” as suggested | Page #17, Line 295- 296 |
| 26. Line 320: Consider saying “an unclear process between sample collection and delivery of results” | Thank you for the suggestion. We have revised the wording to 'an unclear process between sample collection and delivery of results' for better clarity | Page #19, Line #334 |
| 27. Lines 337-341: These lines could go to the section above. | Thank you for the suggestion. We have moved the mentioned lines- “*So women should also know how to use it. They should be able to maintain sterility, that is important too. They should know about the number of hours within which the sample should be taken to the lab, know about the temperature they have to maintain while transporting the sample from home to the lab. All of this should be managed and people should be aware. –(Gynacologist-4)* to the section above as recommended for better organization and flow. | Page#19, Lines #344- 348 |
| 28. Line 329: This could read, “Education about processes around sample collection | Thank you for the feedback. The issue of accuracy and quality will be diluted and the paper really wants to highlight the accuracy and quality of sampling process. | Page #19, Line #350 |
| 29. Lines 364-367: Same quotation as in lines 302-306. This time it was by Gynaecologist 1 and not 3. | Thank you for your observation. The first line was included by mistake, so we have deleted it to avoid repetition and maintain clarity. The correct quotation from Gynaecologist 1 remains in the text. I appreciate your attention to this issue. | Page#18, Line #316 |
| 30. Lines 517-518: Is DHOS not local government. If not, then what is DHOS? This seems contradictory.   . | Thank you for your query. The Department of Health Services (DoHS) in Nepal is a central-level government body under the Ministry of Health and Population (MOHP). It is responsible for implementing national health policies and programs. It is not part of the local government but works in coordination with local authorities to deliver health services across the country |  |
